# Physical activity levels and associated factors among Rohingya older adults living in the refugee camp in Bangladesh

**DOI:** 10.1371/journal.pgph.0006982

**Published:** 2026-07-31

**Authors:** Sabuj Kanti Mistry, Afsana Anwar, Tahera Ahmed, Uday Narayan Yadav, Satyajit Kundu, Shovon Bhattacharjee, Oli Ahmed, Mahesh Kumar Dev, A. R. M. Mehrab Ali, Amir Hossain, Grace McKeon, Simon Rosenbaum

**Affiliations:** 1 ARCED Foundation, Dhaka, Bangladesh; 2 School of Population Health, University of New South Wales, Sydney, Australia; 3 Department of Public Health, Daffodil International University, Dhaka, Bangladesh; 4 Discipline of Psychiatry and Mental Health, School of Clinical Medicine, University of New South Wales, Sydney, Australia; 5 The Human Development and Community Wellbeing, The Kids Research Institute, Perth, Western Australia, Australia; 6 National Centre for Epidemiology and Population Health, The Australian National University, Canberra, Australia; 7 International Centre for Future Health Systems, University of New South Wales, Sydney, Australia; 8 School of Medicine and Dentistry, Griffith University, Gold Coast Campus, Southport, Queensland, Australia; 9 Eastern Regional Eye Care Programme, Biratnagar Eye Hospital, Biratnagar, Nepal; 10 Society for Health Extension and Development (SHED), Cox’s Bazar, Bangladesh; Augusta University, UNITED STATES OF AMERICA

## Abstract

Physical inactivity increases with age, yet evidence on physical activity (PA) among older adults affected by forced displacement remains scarce, particularly in refugee camp settings. We aimed to assess PA levels and identify associated factors among Rohingya older adults aged ≥60 years living in refugee camps in Southern Bangladesh. A cross-sectional survey was conducted between May and August 2024. PA was assessed using the Physical Activity Vital Sign (PAVS), alongside socio-demographic, health, and lifestyle variables collected via a pre-tested semi-structured questionnaire. in line with WHO guidelines, participants were classified as active (≥150 minutes of moderate to vigorous PA per week) or low active (<150 minutes of moderate to vigorous PA per week). Multivariable Firth penalised logistic regression was used to examine factors associated with meeting PA guidelines. The sample comprised 943 participants (mean age 67.5 years), of whom 70% were aged 60–69 years and 66% were male. Overall, 95% were classified as low active. In adjusted analyses, only older age (≥70 years) was significantly associated with lower odds of meeting PA guidelines (OR 0.31, 95% CI 0.11-0.70). Fewer than one in twenty Rohingya older adults met recommended PA levels. These findings highlight the need for tailored, culturally appropriate PA interventions, with particular attention to adults aged 70 years and older.

## Introduction

The Rohingya people are a persecuted minority who have endured a severe, protracted humanitarian crisis, marked by forced displacement from Myanmar due to military violence and egregious human rights abuses [1]. The United Nations High Commissioner for Human Rights (UNHCHR) has characterized this situation as an example of ethnic cleansing [2]. Since 2017, approximately one million Rohingya refugees have resided in southern Bangladesh, with the majority living in refugee camps in Cox’s Bazar. This mass exodus from Myanmar has created one of the world’s largest and most densely populated refugee settlements [3]. According to a recent UNHCR report, there are nearly one million Rohingya refugees are residing in refugee camps in Bangladesh of which over 39,000 are aged 60 and above [4]. The situation has placed immense strain on resources and infrastructure, leading to challenging living conditions, livelihood, health and education prospects for this vulnerable population [5,6].

Previous research has revealed a complex health landscape among Rohingya refugees in Bangladesh [6,7]. Extreme overcrowding in the camps poses significant challenges in delivering essential services [8], including healthcare, non-communicable diseases (NCDs) [9], mental health and psychosocial support services (MHPSS) [10], and clean water, sanitation and hygiene (WASH) facilities [11]. These living conditions, coupled with limited access to health and social services, have a detrimental impact on overall well-being [12]. This situation is particularly dire for older adults, who often live with chronic health issues and limited mobility, making them especially vulnerable to adverse health outcomes [7,13]. For example, a study conducted in 2021 reported that 52.8% of 1058 Rohingya adults had chronic conditions, with this figure rising to 92.5% among those aged 65 years and older [14]. However, these figures may underestimate the true burden due to limited diagnostic efforts in the camp [7]. Likewise, studies conducted among Rohingya older adults in Bangladesh demonstrated high level of depression [15] and disrupted mental wellbeing [16]. Moreover, as the crisis has transitioned from an emergency phase to a protracted humanitarian situation, the health needs of refugees, particularly the management of chronic diseases, has become increasingly complex [17,18]. This shift underscores the necessity of sustained healthcare interventions to address the growing burden of NCDs and mental health problems in the camps.

Physical activity (PA) is widely recognized for its significant benefits on health and well-being, including improved cardiovascular health, enhanced mental well-being, and reduced risk of chronic diseases [19,20]. In various settings, PA has been shown to promote quality of life, boost cognitive function, and support social cohesion [21]. In humanitarian settings, where populations face heightened physical and psychological stress, PA can play a crucial role in promoting resilience, mental health, and overall well-being [22–25]. Growing evidence has shown that PA can simultaneously improve both mental and physical health outcomes [26], effectively reducing symptoms of depression [27], anxiety [28], and cognitive impairment [29]. In addition, incorporating PA into routine care is both essential and ethical, given the strong evidence supporting the benefits of exercise for older adults who are frail [30]. Therefore, cost-effective ways of delivering group interventions like PA that simultaneously target both physical and mental health are essential [31], especially for older adults.

A qualitative study by Wells et al [31] proposed that PA is a feasible and community-endorsed strategy for promoting psychosocial well-being among Rohingya refugees [31]. Likewise, another qualitative study conducted among Rohingya refugees in Bangladesh explored the potential role of sports and PA in psychosocial interventions among adolescents [32]. However, these studies did not identify the prevalence and factors associated with PA among older adults, which is essential to develop targeted physical activity interventions to enhance overall health and well-being. To date, only a single study by Rahman and colleagues has assessed PA quantitatively in the Rohingya context, as part of the investigation of NCD risk factors [9]. This study [9] was conducted among 248 Rohingya refugees aged 18 or above and reported that overall, 89.6% of them were insufficiently active; however, separate data for older adults aged 60 years or over was not reported. As a result, there remains a significant gap in understanding the PA levels, and its determinants for older Rohingya refugees, highlighting the need for further research in this area.

Prior research has commonly used instruments such as the International Physical Activity Questionnaire and the Global Physical Activity Questionnaire to assess PA the population level [33]. While these tools have been validated across a range of settings, they can be challenging to administer in complex humanitarian contexts, particularly among populations with limited health literacy, due to recall bias, cultural differences and misinterpretation of questionnaire items [34]. These challenges are amplified among older adults for whom lengthy or cognitively demanding instruments may further compromise data quality [35]. In this context, brief and pragmatic measures such as the Physical Activity Vita Sign (PAVS) offer a feasible alternative [36]. The PAVS has been widely used in clinical and community settings as a low-burden screening tool, with demonstrated acceptable validity for classifying individuals according to whether they meet recommended PA levels [36–38]. Its simplicity makes it particularly suited to resource-constrained and humanitarian settings where more complex epidemiological instruments may be impractical.

Therefore, this study aimed to assess PA levels using the PAVS tool and identify factors associated with insufficient PA among Rohingya older adults, with the goal of informing contextually appropriate strategies to promote PA in older populations affected by forced displacement.

## Methods

### Ethics statement

The study protocol was approved by the Research Ethics Committee of the Faculty of Health and Life Sciences (FHLS), Daffodil International University, Bangladesh (Ref: FHLS-REC/DIU/2024/0001). Written permission to enter the camps and conduct the survey was obtained from the Office of the Refugee Relief and Repatriation Commissioner. Both verbal and written informed consent were obtained from the participants before administering the survey. Thumbprints were taken from participants unable to read and write after receiving informed consent from their legal guardian(s). The participants could opt out of the study at any time, and the individuals who did not participate would receive the same treatment as those who participated.

### Study design and participants

This cross-sectional study was conducted between May and August 2024 among older adults aged ≥60 years residing in the Rohingya refugee camps in Bangladesh. The prevalence of Moderate-Vigorous Physical Activity (MVPA) in general community-dwelling older adults, has been reported to be between 2.4 – 83.0% across the studies [39]. Therefore, assuming an unknown prevalence of MVPA of 50% among Rohingya older adults, the required sample size was calculated to be 973, using a 5% margin of error, 95% confidence level, 80% statistical power, and a 75% response rate. A local NGO provided a sampling frame for older adults residing in nine of the 34 sub-camps, from which 973 individuals were randomly selected using computer-generated random numbers.

Individuals were eligible to participate if they were aged ≥60 years and were current residents of the refugee camps. The age of the participants was confirmed by verifying the smart cards issued by the United Nations High Commissioner for Refugees (UNHCR). Individuals were excluded if they were diagnosed with any severe mental health conditions, such as schizophrenia, bipolar mood disorder, dementia/cognitive impairments. We also excluded individuals who were unable to communicate effectively because of their old age or for any other reason.

### Study procedure

A semi-structured questionnaire in Bengali language was used for data collection. Three members of the research team (AA, AMA, SKM) translated the English version of the questionnaire, which was then back translated into English to ensure consistency in meaning and that both versions provided the same responses. The Bengali version was then piloted with a small sample (n = 10) of Rohingya older adults to refine its language. However, no modification in language was deemed necessary. We recruited a total of 39 volunteers who were local residents with prior experience in administering health surveys on electronic platforms, and they were supervised by 11 supervisors from the participating NGO. A three-day intensive training session, facilitated by experienced research team members, covered study tools, ethical considerations, and data collection protocols. Face-to-face interviews lasting approximately 30 minutes were conducted using the SurveyCTO mobile platform (https://www.surveycto.com/).

### Measures

#### Outcome measure.

We used the PAVS tool, developed by Greenwood and colleagues [36] to assess PA levels, which has been widely used among older adults [40]. Participants were asked: (1) “On average, how many days per week do you engage in moderate-to-vigorous PA like brisk walking?” and (2) “On those days, how many minutes do you engage in such activities?” These were multiplied to yield the total minutes per week of self-reported MVPA. We then categorised the participants in accordance with the WHO’s Guidelines as ‘active’ (≥150 minutes of MVPA per week) or ‘low active’ (<150 minutes of MVPA per week) [41].

#### Explanatory variables.

Explanatory variables were selected based on a review of relevant literature [15,16,32], suggestions of experts and prior research experience of the authors in this setting. The variable selection was guided by the socio-ecological framework [42] which demonstrates that health outcomes are associated with factors at both individual and societal (community, organization and institutional) levels. The following variables were included:

Demographic characteristics: Age (categorized as 60–69 or ≥70 years), sex (male/female), marital status (married/widowed, separated or unmarried), and education level (formal/no formal education).

Socioeconomic factors: Family size (≤4 or >4 members), living arrangement (alone/with family), and household income source (aid only/aid plus other sources).

Health-related and lifestyle factors: Tobacco use (defined as use of tobacco or smokeless tobacco within the past 30 days [43]), feelings of loneliness (measured using the validated UCLA Loneliness Scale [44]), and self-reported chronic diseases (e.g., hypertension, heart disease, diabetes).

Self-reported data on pre-existing NCDs, including hypertension, heart disease, hypercholesterolemia, diabetes, chronic respiratory diseases, and arthritis-were gathered by asking participants whether they had ever been diagnosed with any of these conditions by a healthcare provider.

The UCLA Loneliness scale [44] has three questions: how often do you feel: (i) lack of companionship, (ii) left out, and (iii) isolated in the last two weeks? Each item is measured with 3-item Likert responses: hardly ever (1 point), some of the time (2 points), and often (3 points). The participants were considered having feelings of loneliness if they answered ‘some of the time’ or ‘often’ to any of the item [45]. This scale has been used previously among the Bangladeshi population [46].

#### Statistical analysis.

Descriptive statistics were summarised using frequencies and percentages. Associations between explanatory variables and PA category were initially explored using chi-square tests. Multivariable associations were examined using Firth penalised logistic regression to identify factors associated with meeting PA recommendations. This approach was selected to address sparse data and separation resulting from the highly imbalanced outcome distribution, and to provide more stable odds ratio estimates through bias-reduced penalised maximum likelihood estimation [47].

Domain-specific exploratory analyses were conducted to examine associations within conceptually related groups of variables. Based on the conceptual characteristics of the variables, predictors were grouped into four domains, and each domain was modelled separately using Firth logistic regression.

Domain 1: (Socio‑demographic and household characteristics) included age group, sex, marital status, family size, living arrangement. Domain 2: (Socioeconomic) comprised education level and household monthly income source. Domain 3: (Lifestyle factors) tobacco use. Domain 4: (Psychosocial wellbeing and health conditions) loneliness and self‑reported chronic conditions, hypertension, heart disease, cholesterol, diabetes, chronic obstructive pulmonary disease (COPD), and arthritis. Domain‑specific results derived from Firth penalised logistic regression were compared with the primary multivariable model to assess consistency in effect direction, magnitude, and statistical significance.

Results are presented as adjusted odds ratios (ORs) with 95% confidence intervals (CIs) and p-values. Analyses were performed in R (Version 4.4.2), with statistical significance set at *p* < 0.05.

#### Patient and public involvement.

Patients and/or the public were not involved in developing research questions, designing, and conducting the study, and disseminating the results.

## Results

### Participants’ characteristics

In this study, out of 973 randomly selected individuals, 943 participants consented to participate, yielding a response rate of 92%. There were no missing data, and 943 participants who consented provided complete data and were included in the final analysis. The sociodemographic characteristics of the 943 participants are summarized in Table 1. The majority were aged between 60–69 years (69.50%), with smaller proportions aged 70 years or older (30.50%), and lived with family members (95.50%). Males constituted 66.30% of the sample, most participants were married (85.70%) and lived in households with four or more members (60.80%). Educational attainment was notably low, with 76.80% of participants lacking any formal education. Additionally, approximately three-quarters of the participants (67.10%) had additional income other than aid for their livelihood. Among the lifestyle and health-related factors, over one-third (38.30%) of the participants reported using tobacco. Additionally, 69.80% of study participants experienced feelings of loneliness. Regarding health-related factors, nearly half of the participants had hypertension (45.40%) and arthritis (46.40%). Other health conditions were less prevalent, including heart disease (25.90%), cholesterol issues (12.80%), COPD (22.90%), and diabetes (16.30%) (Table 1).

**Table 1:** Characteristics of the participants and bivariate analysis (N = 943).

Characteristics		Percentage	Physical activity		
			Low active (%)	Active (%)	*P-value*
Overall			95.30	4.70	
Age groups (years)					
	60 – 69	69.50	94.05	5.95	0.005
	70+	30.50	98.26	1.74	
Sex					
	Male	66.30	94.88	5.12	0.354
	Female	33.70	96.22	3.77	
Marital status					
	Married	85.70	95.17	4.83	0.567
	Widowed, separated and unmarried	14.30	96.3	3.7	
Educational status					
	Having formal schooling	23.20	96.35	3.65	0.417
	No formal schooling	76.80	95.03	4.97	
Family size					
	≤4	39.20	96.49	3.51	0.178
	>4	60.80	94.59	5.41	
Household monthly income source					
	Aid only	23.50	95.05	4.95	0.815
	Aid plus other income	76.50	95.42	4.58	
Living arrangement					
	Living with family	95.50	100	0	0.142
	Living alone	4.50	95.12	4.88	
Using tobacco					
	No	61.70	95.7	4.3	0.493
	Yes	38.30	94.74	5.26	
Feeling of loneliness					
	No	30.20	95.09	4.91	0.813
	Yes	69.80	95.44	4.56	
Hypertension					
	No	54.60	95.73	4.27	0.820
	Yes	45.40	94.86	5.14	
Heart disease					
	No	74.10	97.5	5.29	0.274
	Yes	25.90	96.72	3.28	
Cholesterol					
	No	87.20	95.5	4.5	0.725
	Yes	12.80	94.21	5.79	
Chronic Obstructive Pulmonary Disease (COPD)					
	No	77.10	95.6	4.4	0.756
	Yes	22.90	94.44	5.56	
Diabetes					
	No	83.70	95.69	4.31	0.495
	Yes	16.30	93.51	6.49	
Arthritis					
	No	53.80	96.06	3.94	0.056
	Yes	46.20	94.5	5.5	

### Level of physical activity

Nearly the entire sample (95.3%) were classified as low active (<150 minutes of MVPA per week), with only 4.7% meeting recommended PA levels (≥150 minutes/week; Table 1). In bivariate analysis, age was significantly associated with PA category (p = 0.005). Among individuals aged 60–69 years, only 5.59% met PA recommendations, compared with only 1.74% among those aged ≥70 years. Correspondingly, 94.04% and 98.26% of participants in these age groups respectively were classified as low active. No significant differences in PA levels were observed by sex (p = 0.354), or marital status (p = 0.567). Formal education, family size, and living arrangement were also not significantly associated with PA category (all p’s > 0.056). Similarly, tobacco use and feelings of loneliness did not differ by PA status (p = 0.493 and p = 0.813, respectively). The association between arthritis and PA level approached statistical significance (p = 0.056), with a higher proportion of those with arthritis classified as low active. Overall, age was the only variable significantly associated with PA category in bivariate analysis, while other sociodemographic, lifestyle and health-related factors showed no significant associations.

### Factors associated with physical activity

In the diagnostics for Firth penalised logistic regression models, the absence of multicollinearity was confirmed by a Variance Inflation Factor (VIF) value of less than 10 for each included variable (S1 File). Additionally, the Area Under the Receiver Operating Characteristic curve (AUROC) value of 0.698 indicates an acceptable discriminative ability of the Firth logistic regression model to distinguish between the categories of the dependent variable (S2 File).

Multivariable Firth penalised logistic regression analyses are presented in Table 2. Age was the only factor significantly associated with PA. Individuals aged 70 years and above had significantly lower odds of meeting PA recommendations compared with those aged 60–69 years (OR = 0.31, 95% CI = 0.11–0.70, *p* = 0.0037).

**Table 2:** Factors associated with physical activity among the participants (N = 943).

Characteristics		OR	95% CI	*P*
Age groups (years)				
	60 – 69	*Ref*		
	70+	0.31	0.11-0.7	0.0037
Sex				
	Male	*Ref*		
	Female	0.74	0.33-1.54	0.433
Marital status				
	Married	*Ref*		
	Without partner	1.35	0.43-3.63	0.586
Education level				
	Having formal schooling	*Ref*		
	No formal schooling	1.46	0.7-3.39	0.329
Family size				
	≤4	*Ref*		
	>4	1.31	0.68-2.66	0.431
Household monthly income source				
	Aid plus other income	*Ref*		
	Aid only	1.14	0.51-2.72	0.761
Living arrangement				
	Living alone	*Ref*		
	Living with family	2.72	0.32-3.35	0.43
Using tobacco				
	No	*Ref*		
	Yes	1.15	0.61-2.15	0.657
Feeling of loneliness				
	No	*Ref*		
	Yes	0.9	0.47-1.81	0.762
Hypertension				
	No	*Ref*		
	Yes	1.31	0.68-2.52	0.42
Heart disease				
	No	*Ref*		
	Yes	0.47	0.19-1.04	0.0648
Cholesterol				
	No	*Ref*		
	Yes	0.99	0.33-2.65	0.991
Chronic Obstructive Pulmonary Disease (COPD)				
	No	*Ref*		
	Yes	1.34	0.58-2.91	0.479
Diabetes				
	No	*Ref*		
	Yes	1.66	0.73-3.5	0.217
Arthritis				
	No	*Ref*		
	Yes	1.39	0.73-2.65	0.315

Sex was not significantly associated with PA, although females had lower odds of being physically active than males (OR = 0.74, 95% CI = 0.33–1.54, *p* = 0.433). Marital status was also not associated with PA, with individuals without a partner showing slightly higher odds of being active compared to married individuals (OR = 1.35, 95% CI = 0.43–3.63, *p* = 0.586).

Education level was not significantly associated with PA, although individuals without formal schooling had higher odds meeting PA recommendations compared with those with formal education (OR = 1.46, 95% CI = 0.70–3.39, *p* = 0.329). Family size was similarly not associated with PA, with no significant difference observed between individuals from larger families (> 4 members) versus smaller (≤ 4 members) families (OR = 1.31, 95% CI = 0.68–2.66, *p* = 0.431).

Household income source, living arrangement, tobacco use, and feelings of loneliness were not significantly associated with PA (all *p’s* > 0.05). Among health-related factors, hypertension, cholesterol, COPD, diabetes, and arthritis showed no significant associations. Individuals with heart disease appeared less likely to be physically active, although this association did not reach statistical significance (OR = 0.47, 95% CI = 0.19–1.04, *p* = 0.065).

Domain-specific exploratory analyses were conducted with predictors grouped into four domains based on conceptual characteristics (S3 File). The results were consistent with the primary multivariable model presented in Table 2. Participants aged 70 years or older had significantly lower odds of being physically active (OR = 0.31, 95% CI 0.11–0.71, p = 0.004), while no other socio‑demographic, socioeconomic, lifestyle, psychosocial, or health-related factors were significantly associated with PA.

## Discussion

The present study examined levels of PA and associated factors among Rohingya older adults living in refugee camps in Bangladesh. Overall, PA levels were extremely low, with fewer than one in twenty meeting the WHO recommendation of at least 150 mins of moderate to vigorous PA per week. This finding highlights a substantial gap between recommended and observed PA levels in this population and underscores the need for contextually appropriate strategies to support active aging in humanitarian settings.

Low PA among older adults is a well-documented global concern [19,20,22,23,48–53], and our findings align with prior evidence showing particularly low PA levels among older populations affected by forced displacement [54]. Adequate PA is critical for healthy aging, contributing to the maintenance of mobility and independence, prevention of functional decline [51,52]. In refugee settings, however, opportunities for PA are often constrained by environmental, social and structural barriers, including overcrowding, limited access to safe spaces, adverse weather conditions, restricted mobility and competing survival priorities [55–58]. These barriers are well documented within the Rohingya camps, where older adults frequently experience poor living conditions, chronic health problems and limited access to age-appropriate services [13,59–61]. The convergence of these factors likely contributes to the very low levels of PA observed in this study.

Age was the only factor consistently significantly associated with PA in both bivariate and multivariate analyses, with adults aged 70 years and older substantially less likely to meet PA recommendations than those aged 60–60 years. This finding is consistent with previous research showing declining PA with advancing age [62–64]. Age-related physiological changes, including reductions in muscle mass, strength, and aerobic capacity are known to limit physical functioning and participation in PA among older adults [65–67]. In addition, fear of falls, perceived physical limitations and beliefs about being “too old” to be active may further reduce PA engagement in later life [68]. In humanitarian contexts, these age-related challenges may be compounded by limited access to supportive environments or tailored PA opportunities, placing the oldest adults at particular risk of inactivity [69].

In contrast, other sociodemographic, socioeconomic, lifestyle, psychosocial and health-related factors were not significantly associated with PA levels in multivariable analyses. While differences by sex, education, family size and selected chronic conditions have been reported in other populations [54,70–74], these associations were not evident in this study after accounting for age and other covariates. This may reflect the overwhelming influence of structural and environmental constraints within the camp setting, which may limit PA opportunities across subgroups and reduce variability in behavior. It is also possible that limited statistical power, measurement constraints or the homogeneity of living conditions contributed to the lack of observed associations.

### Implications for policy and practice

Taken together, the findings of the study suggest that PA promotion efforts in Rohingya refugee camp should prioritize older adults aged 70 years and above, who appear to be at significant risk of inactivity. Interventions should be culturally appropriate, feasible within resource-constrained environments, and sensitive to age-related functional limitations. Given the pervasive low levels of PA observed, strategies that focus on maintaining mobility through light-intensity activity, walking, and socially supported movement may be particularly relevant.

In the first place, the organizations and programs prioritizing and working with the Rohingya older adults should play a vital role in promoting PA among older adults in the camps. Only two organizations, Young Power in Social Action (YPSA) [59] and HelpAge International [75] are working with Rohingya older adults in a limited number of sub-camps. They currently do not offer any PA interventions for older adults in the camp except for some recreational activities through age-friendly spaces within the community. So, to address this gap, they should introduce age-friendly, gender and culture-sensitive, and socially supported community-based PA interventions for the older adults in the camps. Interventions should also focus on improving the knowledge and awareness related to PA and its potential benefits among older adults living in the camps. Community-based PA education programs [76] can be delivered using existing community health workers [17] to motivate older adults to engage themselves in appropriate and required PA. Simultaneously 8-week exercise and sports [77] or 10-week PA interventions [78] can be implemented to improve the PA levels in the Rohingya older adults. Future research should explore context-specific barriers and facilitators to PA using longitudinal and qualitative approaches to better inform the design of interventions that can be realistically implemented in humanitarian settings.

### Strengths and limitations of the study

This work provides novel evidence on PA levels among Rohingya older adults living in refugee camps in Bangladesh, a population that has been largely overlooked in prior research. By focusing specifically on older adults in a humanitarian setting, the study offers valuable insights to inform policy makers, implementing agencies, and practitioners working with displaced populations. A key strength was the involvement of trained volunteers who shared language and cultural backgrounds with participants, which likely enhanced trust, cultural sensitivity and the accuracy of data collection. The relatively large sample size further strengthens the robustness of the findings.

Several limitations should be considered. First, the cross-sectional design precludes causal inference and limits interpretation to observed associations. Second, the study was conducted in selected sub-camps, which may restrict the generalizability of findings to the broader Rohingya camp population. Third, physical activity was assessed using the Physical Activity Vital Sign (PAVS), which, while pragmatic and low burden, has not been formally validated in this population and may be subject to recall or social desirability bias. Similarly, chronic health conditions were self-reported, which may have led to underreporting or misclassification, particularly in a population with limited health literacy. Finally, individuals with severe mental health conditions or communication difficulties were excluded, which may introduce selection bias and result in underestimation of inactivity in the most vulnerable subgroups.

## Conclusions

This study highlights extremely low levels of PA among Rohingya older adults living in refugee camps, with fewer than one in twenty meeting recommended PA levels. Advancing age, particularly among adults aged 70 years and older, was the primary factor associated with lower PA, underscoring the heightened vulnerability of the oldest adults in humanitarian settings. By generating context-specific evidence from an under-researched population, this study provides a foundation for informing future PA promotion efforts among older displaced adults. Interventions should prioritize age-appropriate, culturally sensitive and feasible approaches that can be delivered within resource-constrained camp environments. Future research should incorporate longitudinal and qualitative methods to better understand barriers and facilitators to PA in humanitarian contexts and to guide the development of scalable, place-based interventions that support healthy ageing among older refugees.

## Supporting information

S1 File Multicollinearity diagnosis result. (DOCX)

S2 File ROC Curve for Multivariate Logistic Regression model. (DOCX)

S3 File Domain-specific exploratory analysis. (DOCX)

S4 File Study data. (XLSX)
