# Supplementary material for: Physical activity levels and associated factors among Rohingya older adults living in the refugee camp in Bangladesh
Source: PLOS Glob Public Health. 2026 Jul 31;6(7):e0006982. doi: 10.1371/journal.pgph.0006982 (PMC13426958; doi:10.1371/journal.pgph.0006982)
Supplement: S1 File — (DOCX) [file pgph.0006982.s001.docx]

**S1 File: Multicollinearity diagnosis result**

| Variable | VIF |
| --- | --- |
| Age groups (years) | 1.02619 |
| Sex | 1.306425 |
| Marital status | 1.285481 |
| Education level | 1.047183 |
| Family size | 1.087227 |
| Household monthly income source | 1.438468 |
| Living arrangement | 1.002538 |
| Using tobacco | 1.078034 |
| Feeling of loneliness | 1.102461 |
| Hypertension | 1.183943 |
| Heart disease | 1.192594 |
| Cholesterol | 1.572226 |
| Chronic Obstructive Pulmonary Disease (COPD) | 1.572226 |
| Diabetes | 1.179623 |
| Arthritis | 1.131471 |
