# Supplementary figures and images for: Physical activity levels and associated factors among Rohingya older adults living in the refugee camp in Bangladesh
Source: PLOS Glob Public Health. 2026 Jul 31;6(7):e0006982. doi: 10.1371/journal.pgph.0006982 (PMC13426958; doi:10.1371/journal.pgph.0006982)

**S2 File: ROC Curve for Multivariate Logistic Regression model**


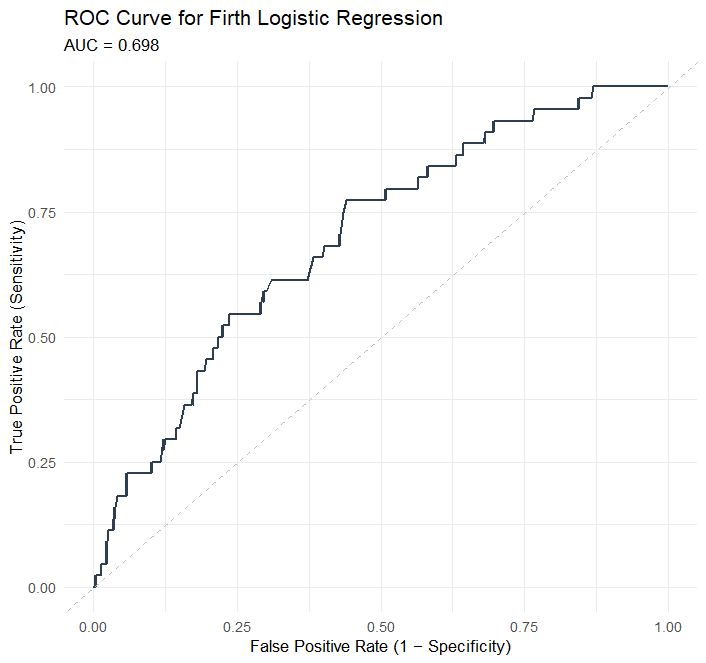

Supplement: S2 File — (DOCX) [file pgph.0006982.s002.docx]
