# Supplementary material for: Physical activity levels and associated factors among Rohingya older adults living in the refugee camp in Bangladesh
Source: PLOS Glob Public Health. 2026 Jul 31;6(7):e0006982. doi: 10.1371/journal.pgph.0006982 (PMC13426958; doi:10.1371/journal.pgph.0006982)
Supplement: S3 File — (DOCX) [file pgph.0006982.s003.docx]

**S3 File: Domain-specific exploratory analysis**

| Characteristics | OR | 95% CI | P | | |
| --- | --- | --- | --- | --- | --- |
| **Socio Demographic and household characteristics** |  |  |  | | |
| Age groups (years) |  |  |  | | |
| 60 – 69 | *Ref* |  |  | | |
| 70+ | 0.31 | 0.11-0.71 | | 0.004 | |
| Sex |  |  |  | | |
| Male | *Ref* |  | | |  |
| Female | 0.82 | 0.37-1.69 | | | 0.61 |
| Marital status |  |  | | |  |
| Married | *Ref* |  | | |  |
| Without partner | 1.31 | 0.43-3.47 | | | 0.62 |
| Family size |  |  | | |  |
| ≤4 | *Ref* |  | | |  |
| >4 | 1.29 | 0.68-2.59 | | | 0.44 |
| Living arrangement |  |  | | |  |
| Living alone | Ref |  | | |  |
| Living with family | 3.15 | 0.38-4.09 | | | 0.35 |
| **Socio economic characteristics** |  |  | | |  |
| Education level |  |  | | |  |
| Having formal schooling | *Ref* |  | | |  |
| No formal schooling | 1.31 | 0.64-3.01 | | | 0.48 |
| Household monthly income source |  |  | | |  |
| Aid plus other income | Ref |  | | |  |
| Aid only | 0.93 | 0.48-1.93 | | | 0.831 |
| **Lifestyle factor** |  |  | | |  |
| Using tobacco |  |  | | |  |
| No | Ref |  | | |  |
| Yes | 1.24 | 0.67-2.27 | | | 0.48 |
| **Psychosocial wellbeing and health conditions** |  |  | | |  |
| Feeling of loneliness |  |  | | |  |
| No | *Ref* |  | | |  |
| Yes | 0.86 | 0.45-1.7 | | | 0.652 |
| Hypertension |  |  | | |  |
| No | *Ref* |  | | |  |
| Yes | 1.21 | 0.64-2.29 | | | 0.556 |
| Heart disease |  |  | | |  |
| No | *Ref* |  | | |  |
| Yes | 0.51 | 0.21-1.1 | | | 0.0898 |
| Cholesterol |  |  | | |  |
| No | *Ref* |  | | |  |
| Yes | 1.16 | 0.43-2.83 | | | 0.762 |
| Chronic Obstructive Pulmonary Disease (COPD) |  |  | | |  |
| No | *Ref* |  | | |  |
| Yes | 1.26 | 0.56-2.66 | | | 0.566 |
| Diabetes |  |  | | |  |
| No | *Ref* |  | | |  |
| Yes | 1.55 | 0.7-3.2 | | | 0.268 |
| Arthritis |  |  | | |  |
| No | *Ref* |  | | |  |
| Yes | 1.38 | 0.74-2.59 | | | 0.304 |
